# Chronic asymptomatic dislocation of a total hip replacement: a case report

**DOI:** 10.4076/1752-1947-3-8956

**Published:** 2009-08-19

**Authors:** Surjit Lidder, Vijai S Ranawat, Nitran S Ranawat, Tudor L Thomas

**Affiliations:** 1 The London Sarcoma Unit, Royal National Orthopaedic Hospital, Stanmore, UK; 2 Department of Orthopaedics and Trauma, Barnet Hospital, Barnet, UK; 3 Department of Orthopaedics and Trauma, Essex Rivers Health Care NHS Trust, Colchester, Essex, UK

## Abstract

**Introduction:**

Dislocation of a prosthetic hip is the second most common complication after thromboembolic disease in patients undergoing total hip arthroplasty, with an incidence reported as 0.5 to 20%. Although the period of greatest risk for dislocation has been reported to be within the first few months after surgery, late dislocation occurs more commonly then previously thought.

**Case presentation:**

A 60-year-old man underwent a right Exeter cemented total hip replacement and was subsequently discharged after appropriate follow-up. He next presented 8 years later complaining of pain in the left groin. An anterioposterior radiograph of the pelvis revealed degenerative changes in the left hip and a dislocated right total hip replacement. The dislocated femoral component had formed a neoacetabulum within the ilium, in which it was freely articulating. He remained pain-free on this side, had 5 cm of true leg length shortening with a good range of movement and was very pleased with his hip replacement. He was later placed on the waiting list for a left total hip replacement.

**Conclusion:**

This case illustrates that a dislocated total hip replacement may occasionally not cause symptoms that cause significant discomfort or reduction in range of movement. The prosthetic femoral head can form a neoacetabulum allowing a full range of pain-free movement. Furthermore it emphasises that with an increased trend to earlier hospital discharge and shorter follow-up, potential complications may be missed. We urge a low index of suspicion for potential complications and suggest that regular review with radiographic follow-up should be made.

## Introduction

Dislocation of the prosthetic hip is the second most common complication secondary to thromboembolic disease in patients undergoing total hip arthroplasty. Studies have reported a widely ranging incidence of 0.5 to 20% [1], with the highest risk believed to be within the first three months after surgery [2]. Few studies have reported the cumulative long-term risk or incidence of late hip dislocation, which is actually greater than previously thought.

We report an unusual case of a long-standing, but asymptomatic, dislocated total hip replacement presenting 8 years after initial surgery. A Medline and PubMed search of the literature reveals that this has not been previously reported.

## Case presentation

A 60-year-old man, referred to the orthopaedic outpatients department in July 1997, presented with a painful right hip of several months' duration. Examination revealed a grossly reduced range of movement in the right hip. His previous medical history included gout, controlled by 300 mg allopurinol. Radiographs of the pelvis revealed severe osteoarthritic changes of the right hip and a normal left hip.

He was kept under review for one year, during which his pain increased and in August 1998 he underwent a right Exeter cemented total hip replacement via a posterior approach. Immediate postoperative recovery until hospital discharge was uneventful, with radiographs of the hip showing no problems. At a routine 6-week follow-up it was noted that although pain free he was making slow progress. There was no leg length discrepancy and the range of movement of the right hip was good, with 100° of flexion, 30° of abduction, 15° of internal rotation and 20° of external rotation. However, the muscular strength was reduced in comparison with his left hip and he had an unsteady gait. He was referred to a physiotherapist and made good progress with improvement in hip strength. Follow-up at one year revealed him to be making excellent progress, and at two years post-operatively he was discharged, entirely symptom free and very happy with his surgical result.

**Figure 1:**
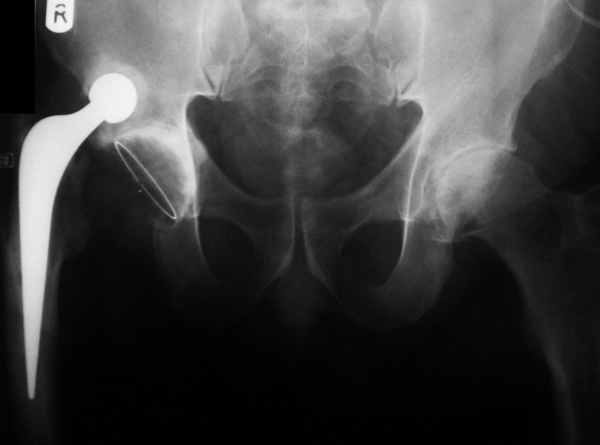
**An anterioposterior radiograph of the pelvis showing degenerative changes of the left hip and the dislocated right Exeter total hip replacement, with the prosthetic femoral head articulating freely within a neoacetabulum**.

**Figure 2:**
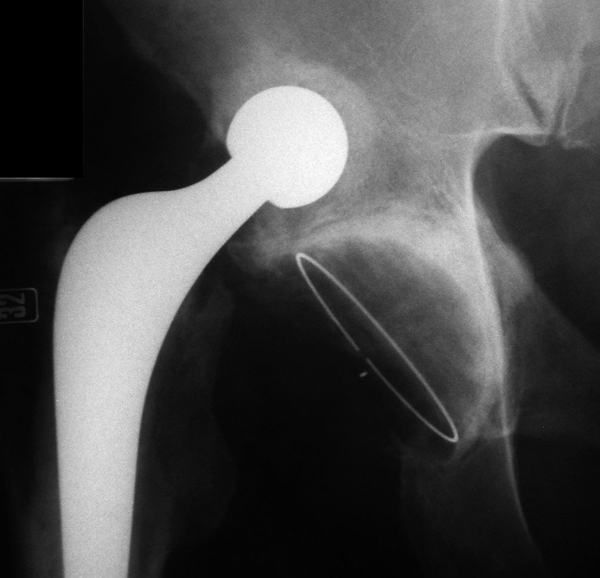
**A magnified view of the dislocated right Exeter total hip replacement, with the prosthetic femoral head articulating freely within a neoacetabulum**.

**Figure 3:**
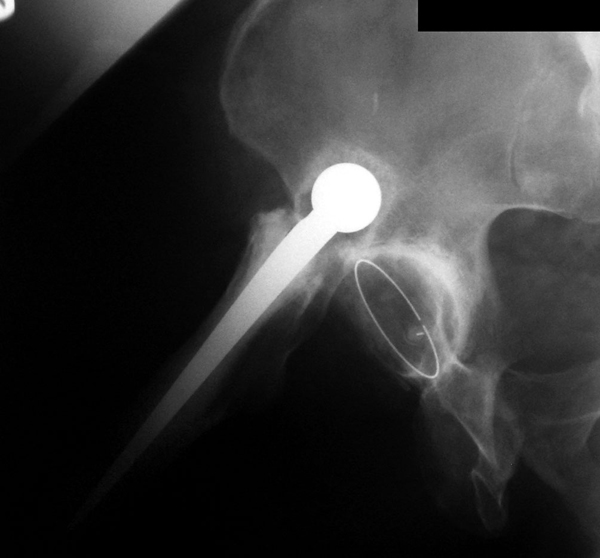
**A radiograph of the right lateral hip showing the dislocated right Exeter total hip replacement, with the prosthetic femoral head articulating freely within a neoacetabulum**.

The patient did not see his general practitioner about hip pain until he next presented in November 2006 complaining of pain in the left groin. An anterioposterior radiograph of the pelvis revealed degenerative changes in the left hip and a dislocated right total hip replacement (Figure 1). The dislocated femoral component had formed a neoacetabulum within the ilium, in which it was freely articulating (Figures 2 and 3). He remained pain free on this side, had 5 cm of true leg length shortening, with a good range of movement, and was very pleased with his hip replacement. He was later placed on the waiting list for a left total hip replacement.

## Discussion

Dislocation of total hip replacement performed via the posterior approach has been reported to occur in 5.8% of cases [3]. Patients usually complain of severe pain and an inability to move the affected leg. The highest incidence of hip dislocation occurs within the first three months after surgery [1,2], and few studies have investigated the risk factors and outcomes for late dislocations, namely those occurring after five years.

Von Knoch et al. showed in 2002 that up to 32% of dislocated hips initially dislocate after 5 years of primary arthroplasty [4]. Factors associated with a greater risk include younger age (median 63 years) at primary total hip arthroplasty, and female gender [4,5]. The cumulative risk of first time dislocation is about 1% at one month, increasing by about 1% every 5 years [5].

Early dislocation may be influenced by component malpositioning or impingement of the femur on the pelvis and it often occurs before the patient has gained full muscle control and strength [6]. Late dislocation is associated with polyethylene wear [7], recurrent hip subluxation, increased soft tissue compliance, neurological decline and trauma [5]. There is also a greater risk with underlying diagnoses of osteonecrosis, inflammatory arthritis or a previous hip fracture [5].

With the ongoing implementation of meeting targets for the delivery of care within 18 weeks, guided in the Musculoskeletal Services Framework by the UK Department of Health [8], patients are being discharged sooner from hospital follow-up. Recommendations state that subsequent follow-up need not be made in traditional orthopaedic outpatient clinics but can be made by primary care health professionals (general practitioners and nurses). We urge a low index of suspicion for potential complications after hip arthroplasty such as deep wound infection, thromboembolic disease and dislocation. This is especially important because there is an increased tendency for earlier hospital discharge and shorter hospital follow-up.

## Conclusion

This case illustrates that a dislocated total hip replacement may occasionally not cause symptoms that cause significant discomfort or reduction in range of movement for which a general practitioner or hospital specialist is consulted. The prosthetic femoral head can form a neoacetabulum, allowing a full range of pain-free movement. Furthermore the case emphasises that with an increased trend to earlier hospital discharge and shorter follow-up, potential complications may be missed. We urge a low index of suspicion for potential complications and suggest that regular review with radiographic follow-up should be made.

## Consent

Written informed consent was obtained from the patient for publication of this case report and accompanying images. A copy of the written consent is available for review by the Editor-in-Chief of this journal.

## Competing interests

The authors declare that they have no competing interests.

## Authors' contributions

All of the named authors were involved in the preparation of this manuscript.
